# CT Texture Analysis for Preoperative Identification of Lymphoma from Other Types of Primary Small Bowel Malignancies

**DOI:** 10.1155/2021/5519144

**Published:** 2021-04-02

**Authors:** Shunli Liu, Chuanyu Zhang, Ruiqing Liu, Shaoke Li, Fenglei Xu, Xuejun Liu, Zhiming Li, Yabin Hu, Yaqiong Ge, Jiao Chen, Zaixian Zhang

**Affiliations:** ^1^Department of Radiology, The Affiliated Hospital of Qingdao University, Qingdao, Shandong, China; ^2^Department of Gastrointestinal Surgery, The Affiliated Hospital of Qingdao University, Qingdao, Shandong, China; ^3^GE Healthcare China, Shanghai, China; ^4^Department of Radiology, Yantai Yuhuangding Hospital, Yantai, Shandong, China

## Abstract

**Objectives:**

To explore the application of computed tomography (CT) texture analysis in differentiating lymphomas from other malignancies of the small bowel.

**Methods:**

Arterial and venous CT images of 87 patients with small bowel malignancies were retrospectively analyzed. The subjective radiological features were evaluated by the two radiologists with a consensus agreement. The region of interest (ROI) was manually delineated along the edge of the lesion on the largest slice, and a total of 402 quantified features were extracted automatically from AK software. The inter- and intrareader reproducibility was evaluated to select highly reproductive features. The univariate analysis and minimum redundancy maximum relevance (mRMR) algorithm were applied to select the feature subsets with high correlation and low redundancy. The multivariate logistic regression analysis based on texture features and radiological features was employed to construct predictive models for identification of small bowel lymphoma. The diagnostic performance of multivariate models was evaluated using receiver operating characteristic (ROC) curve analysis.

**Results:**

The clinical data (age, melena, and abdominal pain) and radiological features (location, shape, margin, dilated lumen, intussusception, enhancement level, adjacent peritoneum, and locoregional lymph node) differed significantly between the nonlymphoma group and lymphoma group (*p* < 0.05). The areas under the ROC curve of the clinical model, arterial texture model, and venous texture model were 0.93, 0.92, and 0.87, respectively.

**Conclusion:**

The arterial texture model showed a great diagnostic value and fitted performance in preoperatively discriminating lymphoma from nonlymphoma of the small bowel.

## 1. Introduction

Primary small bowel malignancies (PSBM) are relatively rare, representing less than 3% of all gastrointestinal tract malignancies [1]. Early detection and differential diagnosis of small bowel tumors can be challenging for both clinicians and radiologists because of the nonspecificity of clinical signs and tumor deep location [2].

Although primary small bowel lymphoma (PSBL) accounts for a small proportion of PSBM (approximately 15%), the incidence has been increasing [3]. Surgery is considered to be the first-line treatment for most small bowel tumors, especially malignant and borderline tumors; however, chemotherapy seems to have a survival benefit over surgery alone in treating PSBL [4]. Due to the different management, it is of great significance to improve the imaging approach for preoperative identification of PSBL and other types of PSBM.

Clinically, several modalities are useful to investigate suspected small bowel tumors including capsule endoscopy, computed tomography (CT), or magnetic resonance (MR) enterography or enteroclysis. However, CT imaging is currently recognized as the mostly used and valuable tool for evaluating small bowel masses. In clinical work, the ability of dynamic contrast-enhanced CT imaging to detect and formulate differential diagnosis of intestinal tumors has been well known. Previous studies have investigated the capacity of conventional multiphasic CT imaging and the diagnostic ability of the CT attenuation values for distinguishing different pathological types of small bowel tumors [5–8]. In addition, Yang et al. have reported the value of dual-energy spectral CT imaging and iodine quantification in the preoperative differentiation between PSBL and small bowel adenocarcinoma [9]. However, there are a number of overlaps between CT findings of PSBL and other small bowel tumors. Therefore, conventional CT imaging mainly focuses on subjective and qualitative features and provides limited quantitative parameters for differential diagnosis of small bowel tumors [6].

Recently, the advances in CT texture analysis have improved the processing capacity for tumor heterogeneity at imaging and provide indirect information of the tumor microenvironment within a certain range of quantitative parameters [10]. CT texture analysis reflects the distribution and relationship of pixels in CT images, which could reveal the subtle differences that cannot be recognized by the human eyes and make up for the shortcomings of conventional CT imaging.

The potential use of CT texture analysis has been widely studied in tumor research and demonstrates to be valuable for prognostic prediction in pathological features, overall survival, and treatment response of multiple tumor types [11–14]. Several studies have declared that CT texture analysis may potentially serve as biomarkers for preoperative risk stratification of small bowel gastrointestinal stromal tumors (GIST) [15, 16]. To the best of our knowledge, the diagnostic ability of CT texture analysis has not yet been fully studied in differentiating lymphoma from other types of PSBM.

We aimed to establish preoperative prediction models based on arterial and venous CT images for discriminating patients with PSBL from those with other types of PSBM. We also compared the predictive efficacy of texture models with that of subjective radiological features.

## 2. Materials and Methods

### 2.1. Patients

This retrospective study was approved by the local ethics committee, and the requirement for informed consent was waived.

**Figure 1:**
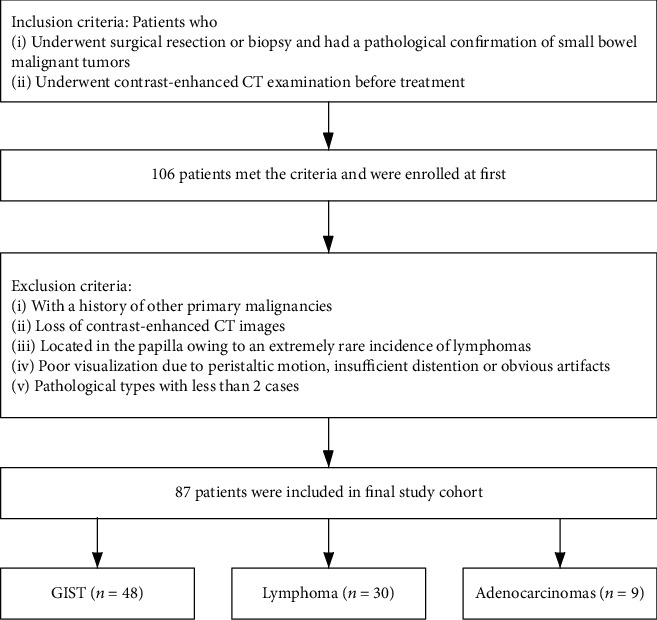
Flowchart of the patient inclusion and exclusion. Data in parentheses are the numbers of patients.

From January 2013 to December 2019, a total of 87 patients with a diagnosis of PSBM who underwent contrast-enhanced CT examination at our hospital were identified and included in this study (Figure 1).

The inclusion criteria were as follows: (1) a pathological confirmation of small bowel malignant tumors based on histological examinations of biopsied or resected tissues and (2) availability of contrast-enhanced CT examination before treatment.

The exclusion criteria were as follows: (1) a history of other primary malignancies (*n* = 1); (2) loss of contrast-enhanced CT images (*n* = 4); (3) located in the duodenal papilla owing to an extremely rare incidence of lymphomas in the ampulla region (*n* = 53) [17]; (4) poor visualization of the lesion due to peristaltic motion, insufficient distention, or obvious artifacts (*n* = 5); and (5) pathological types with less than 3 cases (*n* = 6).

The clinical data, including gender, age, histologic type, and clinical symptoms (melena, abdominal pain, and intestinal obstruction), was obtained and recorded from the electronic medical record system. Patient and tumor characteristics are summarized in Tables 1 and 2.

### 2.2. Image Acquisition and Analysis

CT examinations were performed on a multidetector row scanner (SOMATOM Definition Flash, Siemens Medical Systems; iCT 256, Philips Healthcare; or Optima CT670, GE Healthcare). All patients were requested to fast for at least six hours before the procedure. All patients underwent abdominal CT protocol or CT enterography. For abdominal CT protocol, patients received 600–1000 mL water orally prior to the examination. For CT enterography, patients were encouraged to drink 1000–2000 mL 20% mannitol for over 40–60 min prior to the CT scanning.

All patients were in the supine position, and the scan covered the entire abdomen. The patients were trained to hold their breath during CT scanning. Intravenous 1.0 mL/kg contrast medium (iohexol injection, 300 mg/mL, Beilu Pharmaceutical Co. Ltd., Beijing, China) was injected at a flow rate of 3.0–3.5 mL/s using a power injector (Ulrich CT Plus 150, Ulrich Medical), followed by a saline flush (20 mL). Arterial phase scanning and venous phase scanning were performed at 30 seconds and 70 seconds, respectively, after initiation of contrast material injection.

The CT scanning parameters were as follows: automatic tube current and tube voltage 120 kV, detector collimation 64 × 0.6 or 128 × 0.625 mm, matrix 512 × 512, slice thickness 5 mm, slice interval 5 mm, and reconstructed section thickness 1.25 or 2 mm.

### 2.3. Image Interpretation and Segmentation

Transverse reconstructed CT images were reviewed and interpreted by two abdominal radiologists (reader 1 and reader 2, with 3 and 10 years of working experience, respectively) without knowledge of the clinical data of patients. The radiological features derived from subjective CT interpretation were evaluated by the two radiologists with a consensus agreement. The observed contents were recorded, as follows: (1) location (duodenum, jejunum, or ileum), (2) shape (regular or irregular), (3) margin (well defined or ill defined), (4) lumen dilation (positive or negative), (5) intussusception (positive or negative), (6) enhancement pattern (homogeneous or heterogeneous), (7) enhancement level (mild, moderate, or high), (8) adjacent peritoneum (clear or unclear), and (9) locoregional lymph node (enlarged or non-enlarged). The interpretation criteria were mainly based on clinical experience or previous studies [5, 18]. Lumen dilation was considered to be present if intralesion dilation of the lumen was observed on at least two planes. For the enhancement pattern, masses with intratumoral low-enhancing or nonenhancing areas were considered as heterogeneous enhancement. The high enhancement level was defined as solid components with the CT attenuation greater than 90 HU in arterial CT images. Lesions with CT attenuation less than 60 HU in arterial CT images were considered to be mild enhancement. An enlarged lymph node was considered to be present when a lymph node was greater than 10 mm in a short-axis diameter.

The arterial and venous reconstructed CT images were segmented using ITK-SNAP 3.8.0 (http://www.itksnap.org,USA).The region of interest (ROI) was manually delineated along the edge of the lesion on the largest slice by reader 1, excluding bowel lumen and blood vessels. To evaluate the reproducibility of feature extraction, 30 cases of CT images were randomly selected for calculating inter- and intraclass correlation coefficients (ICCs). Reader 2 independently drew the ROIs of the 30 cases. Reader 1 repeated the segmentations three months later. Texture features with an ICC greater than 0.75 suggested good agreement [19].

### 2.4. Feature Extraction and Selection

The original images were normalized before feature extraction, and texture features were automatically calculated and extracted by using AK software (Analysis Kit 1.0.3; GE Healthcare, China). A total of 402 quantified features were extracted from the delineated ROIs, including 42 histograms, 15 form factor features, 180 gray level run-length matrix (GLRLM) features with an offset of 1/4/7, 154 gray level cooccurrence matrix (GLCM) features with an offset of 1/4/7, and 11 grey level size zone matrix (GLSZM) features. The image analysis and feature extraction were performed separately for the arterial phase and venous phase based on CT images, following the same procedure.

We followed a four-step procedure to identify robust and predictive texture features. First, the texture features with both inter- and intrareader ICCs > 0.75 were retained for further procedure (Supplementary materials Part 1). Second, Mann–Whitney *U* test was performed, exploring whether the features were significantly different between two groups. Then, univariate logistic regression was applied to select related features (with *p* < 0.05). Finally, we used the minimum redundancy maximum relevance (mRMR) algorithm to select the feature subsets; 8 features with high correlation and low redundancy were retained.

### 2.5. Model Construction and Evaluation

Two texture models based on the arterial phase and venous phase were constructed, by using the multivariable logistic regression to filter the independent features and construct the multivariable model, through backward stepwise selection with the likelihood ratio test to select the most predictive feature subset. The radiomics score of each patient was calculated.

Meanwhile, the clinical and radiologic features with *p* < 0.1 in univariate logistic regression were selected to develop a clinical model using the multivariate logistic regression analysis.

**Figure 2:**
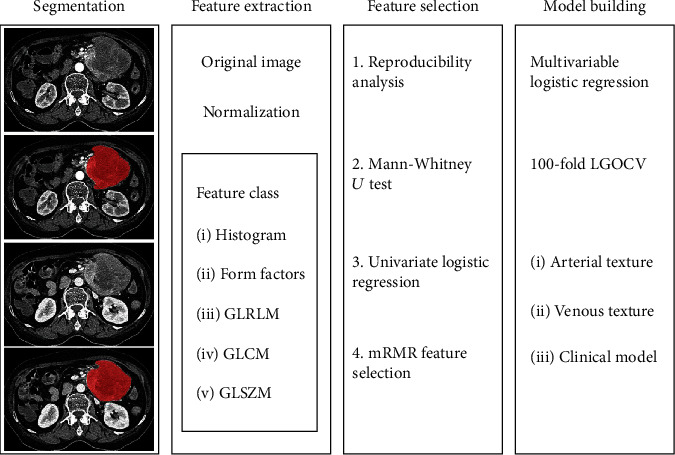
Flowchart of texture analysis. Main steps are tumor segmentation, feature extraction and selection, model construction, and validation. GLRLM: gray level run-length matrix; GLCM: gray level cooccurrence matrix; GLSZM: grey level size zone matrix; mRMR: minimum redundancy maximum relevance; LGOCV: leave group out crossvalidation.

The 100-fold leave group out crossvalidation (LGOCV) was performed to verify that the multivariate models were valuable in discriminating one group from anther group and the result was not due to overfitting. The data analysis workflow is shown in Figure 2.

In order to assess the reliability of CT texture analysis, the support vector machine (SVM) also has been applied further for comparative analysis.

### 2.6. Statistical Analyses

The differences of continuous variables were analyzed by the Mann–Whitney *U* test, and the chi-square test or Fisher's exact test was used for categorical variables. The diagnostic performance of multivariate models was evaluated using ROC analysis and area under the ROC curve (AUC). Diagnostic sensitivity, specificity, accuracy, positive predictive value (PPV), and negative predictive value (NPV) were also calculated. The corresponding definition/equation was presented in Supplementary materials Part 2. All these statistical analyses were performed with R statistical software 3.5.1 (R Foundation for Statistical Computing). A two-tailed *p* value of less than 0.05 was considered statistically significant. To control false-positive rates in multiple testing, the false discovery rate-adjusted *p* values were also calculated during multiple univariate analysis [20].

## 3. Results

### 3.1. Patient Characteristics

A total of 87 lesions from 87 patients were enrolled in the retrospective study for analysis: 48 gastrointestinal stromal tumors (GIST) (2 duodenal, 20 jejunal, and 26 ileal), 30 lymphomas (1 duodenal, 6 jejunal, and 23 ileal), and 9 adenocarcinomas (1 duodenal, 6 jejunal, and 2 ileal). Of these patients, there were 47 males and 40 females, and the mean age was 58 years (age range, 4–80 years).

### 3.2. Clinical and Radiological Feature Evaluation

Our data showed that controlling the false discovery rate in multiple chi-square tests found as many significant results as without false discovery rate adjustment (Table 2). For the clinical data, no significant difference was found between the nonlymphoma group and lymphoma group with regard to the gender (*p* = 0.206) and intestinal obstruction (*p* = 0.177). There was a significant difference between the nonlymphoma group and lymphoma group in the age (*p* = 0.008), melena (*p* = 0.001), and abdominal pain (*p* = 0.008). For radiological features, the location, shape, margin, dilated lumen, intussusception, enhancement level, adjacent peritoneum, and locoregional lymph node differed significantly between the nonlymphoma group and lymphoma group (*p* ≤ 0.001–0.037). However, there was no significant difference between the nonlymphoma group and lymphoma group in the enhancement pattern (*p* = 0.053).

The univariate logistic regression analysis of clinical data and radiological features is shown in Supplementary Table 1. Multivariate logistic regression analysis revealed that the margin, locoregional lymph node, enhancement level, and enhancement pattern were independent indicators to distinguish the nonlymphoma from the lymphoma of the small bowel (Table 3). The ill-defined margin, homogeneous enhancement, mild or moderate enhancement, and enlarged lymph node were apt to be a PSBL rather than other types of PSBM. The sensitivity, specificity, accuracy, and AUC of the clinical model were 93.3%, 79.0%, 83.9%, and 0.93 (95% CI 0.87–0.98), respectively (Table 4).

### 3.3. Texture Feature Evaluation

After dimension reduction, 8 texture features of the arterial phase, 8 texture features of the venous phase, and 8 texture features of the dual phases were selected. There were significant differences of all those texture parameters between small bowel lymphoma and nonlymphoma (Supplementary Tables 2 and 3). The diagnostic performance of the selected texture features is presented in Supplementary Tables 4 and 5.

By using multivariate logistic regression analysis, the arterial texture model was generated using 6 selected features from arterial CT images (Supplementary Table 6). The sensitivity, specificity, accuracy, and AUC were 83.3%, 89.3%, 87.2%, and 0.92 (95% CI 0.87–0.98), respectively (Table 4). Similarly, the venous texture model was developed using 6 selected features (Supplementary Table 7). The sensitivity, specificity, accuracy, and AUC were 73.3%, 85.7%, 81.4%, and 0.87 (95% CI 0.79–0.94), respectively (Table 4). The corresponding RAD scores are shown in Supplementary materials Part 3.

**Figure 3:**
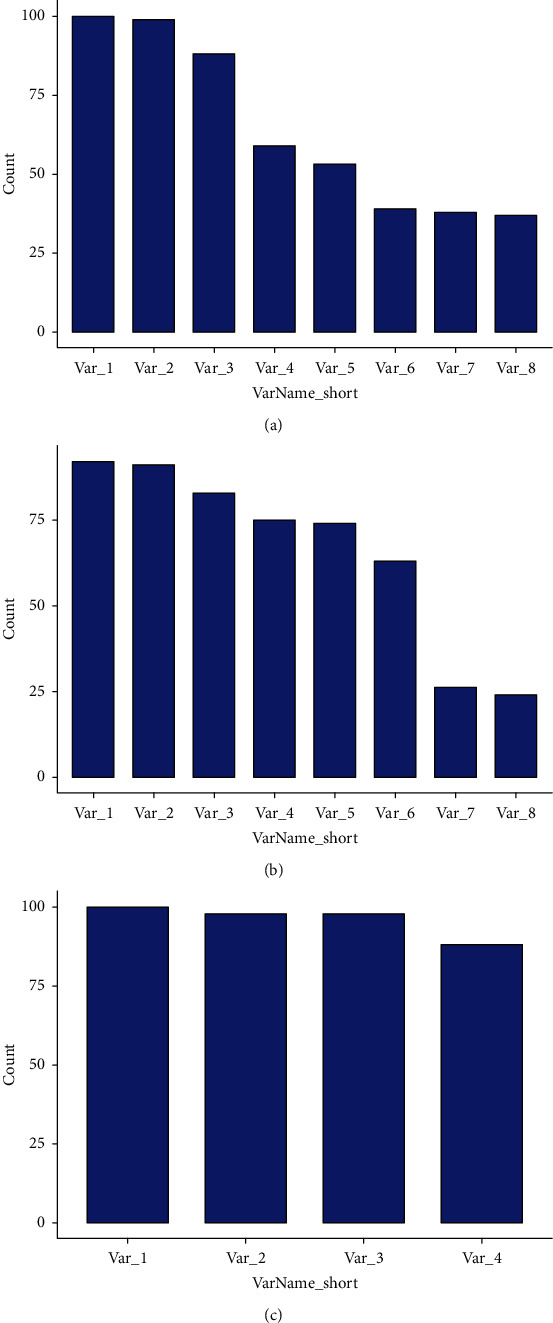
The appeared times of selected features for 100-fold leave group out crossvalidation (LGOCV) in the arterial texture model (a), venous texture model (b), and clinical model (c). The concrete details are shown in Supplementary materials Part 4.

As seen in Table 5, the crossvalidation trials showed that the average accuracy, sensitivity, and specificity values of the three multivariate models were all relatively stable in the training and validation sets. The predictive performance of the arterial texture model was slightly better than that of the venous model and clinical model. The appeared times of selected features during the 100-time crossvalidations are shown in Figure 3. Most texture features and all radiological features appeared more than 50 times during the 100-time trials, which means that these features had high stability and diagnostic values [21].

The diagnostic value of the SVM classification algorithm was presented in Supplementary Table 8. The predictive performance of SVM classifications was slightly better than that of the logistic regression model.

## 4. Discussion

The detection and differential diagnosis of small bowel neoplasms has been attracting the attention of researchers. Our study developed and validated two CT-based texture models and one radiological model as a novel approach to preoperatively differentiate the PSBL from other types of PSBM, which has never been reported previously.

Most previous studies mainly focused on evaluating the morphological findings and enhancement pattern of small bowel neoplasms for differential diagnosis [5, 6]. Our data showed that the clinical features (age, melena, and abdominal pain) and radiological features (location, shape, margin, dilated lumen, intussusception, enhancement level, adjacent peritoneum, and locoregional lymph node) differed significantly between the nonlymphoma group and lymphoma group. As reported previously, lymphoma of the small bowel tended to be more homogenous and show less contrast enhancement compared with other small bowel malignancies [22]. Our findings were relatively consistent with conventional consensus.

In the present study, 2 multivariate texture models, based on arterial and venous phases, were built to aid in preoperatively discriminating PSBL from other types of PSBM. The arterial logistic model showed a better diagnostic value than the venous logistic model; however, the predictive performance of the venous SVM classifier performed slightly better than that of the arterial SVM classifier. According to previous studies, the arterial phase can reflect the blood supply and functional capillary density and the venous phase may reflect more dysfunctional neovessels and represent distribution of contrast media in interstitial spaces [23]. Our findings indicated that the arterial and venous texture features both played a role in reflecting the heterogeneity of small intestine neoplasms.

Our data also suggested that the clinical model has a similar diagnostic ability compared to the arterial texture model. The clinical model is a multivariate logistic regression model enrolling 4 radiological features, including the margin, locoregional lymph node, enhancement level, and enhancement pattern. The results indicated that the ill-defined margin, enlarged lymph node, mild or moderate enhancement, and homogeneous enhancement derived from subjective CT interpretation were independent indicators of a diagnosis of small bowel lymphoma. Attentively, the definition of the enhancement level and enhancement pattern was based on the CT attenuation of small bowel neoplasms in arterial CT images.

Therefore, our findings indicated that the routine radiological features and texture analysis based on arterial CT imaging both held great diagnostic value in distinguishing nonlymphoma and lymphoma of the small bowel. Shinya et al. also reported that the CT attenuation of the arterial phase performed better than that of the venous phase in discriminating small bowel GIST from lymphoma, with an accuracy of 78.6% and 75.0%, respectively [6]. The potential explanation is that the blood supply distribution of small bowel lymphoma might be less robust and more homogeneous than that of GIST and adenocarcinoma of the small bowel.

The results of 100-fold crossvalidation showed that 5 of the selected 6 features in the arterial model appeared more than 50 times during the 100 times crossvalidations and all 6 texture features of the venous model appeared more than 50 times, indicating that the two models were reliable and not overfitting. The arterial texture model included 1 GLRLM feature and 5 GLCM features. GLRLM_RunLengthNonuniformity, GLCM_ClusterShade, and GLCM_ Correlation had great value and stability in identifying small bowel lymphoma. RunLengthNonuniformity describes the similarity of run lengths throughout the image, with a higher value indicating more heterogeneity among run lengths in the image. Cluster shade is a measure of the skewness and uniformity of the GLCM, with a higher value implying greater asymmetry about the mean. Correlation is a value between 0 (uncorrelated) and 1 (perfectly correlated) showing the linear dependency of gray level values to their respective pixels in the GLCM. In the venous texture analysis, 1 histogram feature, 2 GLRLM features, 2 GLCM features, and 1 GLSZM feature formed the multivariable model. GLRLM_RunLengthNonuniformity and GLCM_Correlation also had high stability and diagnostic values in the venous texture model. Besides, GLRLM_HighGreyLevelRunEmphasis and GLSZM_GreyLevelNonuniformity held a certain value in the venous texture model. HighGreyLevelRunEmphasis reflects the distribution of the higher gray level values, with a higher value indicating a greater concentration of high gray level values in the image. GreyLevelNonuniformity evaluates the variability of gray level intensity values in the image, with a lower value indicating more homogeneity in intensity values. Our data suggested that there was significant difference in the heterogeneity of small bowel lymphoma and nonlymphoma and those texture parameters could help to discriminate primary lymphoma from other small bowel malignancies.

The crossvalidation trials showed that the diagnostic efficiency of three multivariate models were all relatively valuable and stable in the training and validation sets. However, the radiological features are a type of subjective findings with individual experience, leading potential diagnostic variation in the imaging analysis. CT texture analysis is an objective tool, and the overall predictive value of our arterial texture model was slightly better than that of the clinical model during crossvalidation trials. The predictive performance of SVM classifications was slightly better than that of the logistic regression model. Hence, CT texture analysis might provide more objective and valuable information in preoperatively discriminating lymphoma from nonlymphoma of the small bowel.

CT texture analysis has been widely investigated in risk grade prediction and prognosis assessment of GIST [15, 16, 24]. The role of CT texture analysis in discriminating lymphoma from nonlymphoma of the small bowel has never been reported previously, which might be due to the rare incidence of PSBL. Several studies have proved the potential promise of CT texture or radiomics analysis in identifying gastric lymphoma. Ma et al. reported that venous CT radiomics analysis had a potential to accurately differentiate Borrmann type IV gastric cancer from primary gastric lymphoma [25]. In another study, Ba-Ssalamah et al. found that CT texture features proved to be highly successful in distinguishing between gastric adenocarcinoma and lymphoma and GIST and lymphoma, with low misclassification [26]. CT texture analysis over small bowel lymphoma still deserves further investigation.

Our study had several limitations. First, it was a retrospective single-center study and the sample size was relatively small and imbalanced. We will further explore the effect of imbalanced data using oversampling or sample weighting method such as SMOTE on the texture analysis model in our future work [27]. Second, bias in patient selection was unavoidable in this retrospective study and no external validation was set owing to small sample size. Hence, the texture model deserves prospective and external validation to confirm its practicability. Third, texture analysis based on delayed-phase CT images was not performed in this study due to no available reconstructed images. The diagnostic efficiency of delayed texture features in small bowel malignant tumors requires further investigation. Finally, the number of patients with small bowel adenocarcinoma was relatively small. Therefore, those patients were grouped together with patients with GIST, as the nonlymphoma group. Further studies with a more precise classification and lager sample size will be needed.

In conclusion, the arterial texture model showed a great diagnostic value and fitted performance in differentiating PSBL from other types of PSBM, which could provide objective information to screen those patients with suspicion of PSBL.

## Figures and Tables

**Table 1:** Clinicopathological characteristics of patients with primary small bowel malignancies.

Feature	*n* = 87 (percentage)
Gender	
Male	47 (54.0%)
Female	40 (46.0%)
Age	
<50 years	18 (20.7%)
≥50 years	69 (79.3%)
Location	
Duodenum	4 (4.6%)
Jejunum	32 (36.8%)
Ileum	51 (58.6%)
Histologic type	
GIST	48 (55.2%)
Lymphoma	30 (34.5%)
Adenocarcinomas	9 (10.3%)

**Table 2:** The univariate analysis of clinical data and radiological features between the nonlymphoma group and the lymphoma group in patients with primary small bowel malignancies.

Feature	Nonlymphoma (*n* = 57)	Lymphoma (*n* = 30)	*p* value	FDR-adjusted *p* values
Gender			0.206	0.050
Male	28	19		
Female	29	11		
Age			0.008	0.029
<50	7	11		
≥50	50	19		
Melena			0.001	0.011
Negative	29	26		
Positive	28	4		
Abdominal pain			0.008	0.029
Negative	28	6		
Positive	29	24		
Intestinal obstruction			0.177	0.046
Negative	53	30		
Positive	4	0		
Location			0.037	0.039
Duodenum	3	1		
Jejunum	26	6		
Ileum	28	23		
Shape			0.002	0.018
Regular	27	4		
Irregular	30	26		
Margin			0.001	0.011
Clear	32	6		
Unclear	25	24		
Dilated lumen			0.002	0.018
Negative	49	17		
Positive	8	13		
Intussusception			0.017	0.036
Negative	56	25		
Positive	1	5		
Enhancement pattern			0.053	0.043
Homogeneous	20	17		
Heterogeneous	37	13		
Enhancement level			<0.001	0.004
Mild	3	12		
Moderate	15	11		
High	39	7		
Adjacent peritoneum			0.002	0.018
Clear	42	12		
Unclear	15	18		
Locoregional lymph node			<0.001	0.004
Nonenlarged	46	7		
Enlarged	11	23		

FDR: false discovery rate.

**Table 3:** The multivariate logistic regression analysis of the clinical data and radiological features.

	Log OR	SE	OR	*p* value
Margin	3.265	1.156	26.179	0.005
Locoregional lymph node	2.984	0.825	19.766	<0.001
Enhancement level	−2.148	0.604	0.117	<0.001
Enhancement pattern	−2.17	0.906	0.114	0.017

Log OR: Logarithm of odds ratio; SE: standard error; OR: odds ratio.

**Table 4:** The diagnostic performance of the clinical model and two texture models.

	Arterial texture	Venous texture	Clinical model
AUC (95% CI)	0.92 (0.87-0.98)	0.87 (0.79-0.94)	0.93 (0.86-0.98)
Accuracy	0.872	0.814	0.839
Sensitivity	0.833	0.733	0.933
Specificity	0.893	0.857	0.790
PPV	0.804	0.730	0.700
NPV	0.910	0.859	0.957

PPV: positive predictive value; NPV: negative predictive value.

**Table 5:** The crossvalidation of three multivariate models.

	Arterial texture		Venous texture		Clinical model	
	Training	Test	Training	Test	Training	Test
Accuracy	0.900	0.831	0.838	0.762	0.854	0.828
Sensitivity	0.877	0.829	0.840	0.780	0.818	0.801
Specificity	0.941	0.833	0.832	0.727	0.920	0.883

## Data Availability

The data underlying the findings of our study are publicly available wherever possible. Please send an email to Zaixian Zhang (email: befate@126.com) if required.

## Abbreviations

CT: Computed tomography
PSBL: Primary small bowel lymphoma
PSBM: Primary small bowel malignancies
GIST: Gastrointestinal stromal tumor
ROI: Regions of interest
ROC: Receiver operating characteristic
AUC: Area under the curve
mRMR: Minimum redundancy maximum relevance
LGOCV: Leave group out crossvalidation
PPV: Positive predictive value
NPV: Negative predictive value.

## Additional Points

*Key points.* Texture features extracted from arterial CT images outperformed those from venous CT images in identifying small bowel lymphoma. The clinical model and arterial texture models held a similar value in discriminating lymphoma from nonlymphoma of the small bowel. The texture models and clinical model showed relatively stable performance during crossvalidation trials.

## Supplementary Materials

Supplementary Materials The inter- and intrareader correlation coefficients (ICCs) are shown in Supplementary materials Part 1. The definition/equation of accuracy, sensitivity, specificity, positive predictive value, and negative predictive value was presented in Supplementary materials Part 2. The arterial and venous texture rad-scores are shown in Supplementary materials Part 3. The appeared times of selected features for 100-fold leave-group-out crossvalidation (LGOCV) in the arterial texture model, venous texture model, and clinical model are shown in Supplementary materials Part 4. The univariate logistic regression analysis of clinical data and radiological features is shown in Supplementary Table 1. The statistical description of the selected arterial and venous texture features is presented in Supplementary Tables 2 and 3. The diagnostic performance of the selected arterial and venous texture features is presented in Supplementary Tables 4 and 5. The multivariate logistic regression analysis of the selected arterial and venous texture features is shown in Supplementary Tables 6 and 7. The diagnostic performance of the SVM classifier and logistic regression model is seen in Supplementary Table 8.
