# Supplementary material for: CT Texture Analysis for Preoperative Identification of Lymphoma from Other Types of Primary Small Bowel Malignancies
Source: Biomed Res Int. 2021 Apr 2;2021:5519144. doi: 10.1155/2021/5519144 (PMC8041543; doi:10.1155/2021/5519144)
Supplement: Supplementary Materials — The inter- and intrareader correlation coefficients (ICCs) are shown in Supplementary materials Part 1. The definition/equation of accuracy, sensitivity, specificity, positive predictive value, and negative predictive value was presented in Supplementary materials Part 2. The arterial and venous texture rad-scores are shown in Supplementary materials Part 3. The appeared times of selected features for 100-fold leave-group-out crossvalidation (LGOCV) in the arterial texture model, venous texture model, and clinical model are shown in Supplementary materials Part 4. The univariate logistic regression analysis of clinical data and radiological features is shown in Supplementary Table 1. The statistical description of the selected arterial and venous texture features is presented in Supplementary Tables 2 and 3. The diagnostic performance of the selected arterial and venous texture features is presented in Supplementary Tables 4 and 5. The multivariate logistic regression analysis of the selected arterial and venous texture features is shown in Supplementary Tables 6 and 7. The diagnostic performance of the SVM classifier and logistic regression model is seen in Supplementary Table 8. [file 5519144.f1.docx]

Part 1

**Intro-class correlation coefficients (ICCs):**

the range of ICC on artery phase: [-0.1263214462650997, 1.0]

the range of ICC on venous phase: [-0.8960471663989416, 1.0]

182 arterial features and 349 venous features showed high reproducibility, with ICCs > 0.75.

**Inter-class correlation coefficients:**

the range of ICC on artery phase: [-0.38830796513621524, 1.0]

the range of ICC on venous phase: [-0.03737531571783226, 1.0]

187 arterial features and 328 venous features showed high reproducibility, with ICCs > 0.75.

Part 2

Schematic table of CT texture analysis in predicting primary small-bowel lymphoma

|  | Non-Lymphoma (pathology) | Lymphoma (pathology) |
| --- | --- | --- |
| Non-Lymphoma (texture) | a | b |
| Lymphoma (texture) | c | d |

The definition/equation of accuracy, sensitivity, specificity, positive predictive value and negative predictive value

Accuracy = (a+d)/(a+b+c+d)

Sensitivity = d/(b+d)

Specificity = a/(a+c)

Positive predictive value = d/(c+d)

Negative predictive value = a/(a+b)

Part 3

**The Rad-score of arterial texture features**

Radscore = -3.38918976954887 * (Intercept) + 3.98340724109274 * Correlation_AllDirection_offset1_SD + 1.58198566146331 * RunLengthNonuniformity_AllDirection_offset4_SD - 2.16621277268356 * ClusterShade_AllDirection_offset4_SD - 4.95942463353464 * ClusterShade_angle90_offset4 + 1.10000697896781 * HaralickCorrelation_AllDirection_offset4_SD - 2.76375802432647 * ClusterProminence_AllDirection_offset7_SD

**The Rad-score of venous texture features**

Radscore = -1.1040090128204*(Intercept) - 0.920118049113411 * GreyLevelNonuniformity_AllDirection_offset4_SD + 3.21784054907659 * RunLengthNonuniformity_AllDirection_offset4_SD - 1.09981700244248 * Percentile90 + 1.02256635009044 * Correlation_AllDirection_offset1_SD - 1.47149151932455 * HighGreyLevelRunEmphasis_AllDirection_offset4_SD - 1.02714269609757 * Correlation_angle45_offset4

Part 4

The appeared times of arterial texture features for 100 folds leave-group-out cross-validation (LGOCV):

| **VarName_short** |  | **VarName** | **Count** |
| --- | --- | --- | --- |
| Var_1 |  | ClusterShade_angle90_offset4 | 100 |
| Var_2 |  | Correlation_AllDirection_offset1_SD | 99 |
| Var_3 |  | HaralickCorrelation_AllDirection_offset4_SD | 88 |
| Var_4 |  | RunLengthNonuniformity_AllDirection_offset4_SD | 59 |
| Var_5 |  | ClusterShade_AllDirection_offset4_SD | 53 |
| Var_6 |  | Maximum3Ddiameter | 39 |
| Var_7 |  | ClusterProminence_AllDirection_offset7_SD | 38 |
| Var_8 |  | ClusterProminence_angle135_offset1 | 37 |

The appeared times of venous texture features for 100 folds leave-group-out cross-validation (LGOCV):

| **VarName_short** |  | **VarName** | **Count** |
| --- | --- | --- | --- |
| Var_1 |  | RunLengthNonuniformity_AllDirection_offset4_SD | 92 |
| Var_2 |  | Correlation_angle45_offset4 | 91 |
| Var_3 |  | Correlation_AllDirection_offset1_SD | 83 |
| Var_4 |  | Percentile90 | 75 |
| Var_5 |  | GreyLevelNonuniformity_AllDirection_offset4_SD | 74 |
| Var_6 |  | HighGreyLevelRunEmphasis_AllDirection_offset4_SD | 63 |
| Var_7 |  | MajorAxisLength | 26 |
| Var_8 |  | HaralickCorrelation_AllDirection_offset4_SD | 24 |

The appeared times of radiological features for 100 folds leave-group-out cross-validation (LGOCV):

| **VarName_short** |  | **VarName** | **Count** |
| --- | --- | --- | --- |
| Var_1 |  | Loco-regional lymph node | 100 |
| Var_2 |  | Enhancement level | 98 |
| Var_3 |  | Margin | 98 |
| Var_4 |  | Enhancement pattern | 88 |

**Supplementary Table 1**. The univariate logistic regression analysis of clinical data and radiological features

| Feature | Log OR | SE | OR | p value |
| --- | --- | --- | --- | --- |
| Age | -0.051 | 0.019 | 0.951 | 0.009 |
| Melena | -1.837 | 0.599 | 0.159 | 0.002 |
| Abdominal pain | 1.351 | 0.528 | 3.862 | 0.010 |
| Location | 0.989 | 0.454 | 2.687 | 0.029 |
| Shape | 1.766 | 0.599 | 5.850 | 0.003 |
| Margin | 1.633 | 0.529 | 5.120 | 0.002 |
| Dilated lumen | 1.544 | 0.530 | 4.684 | 0.004 |
| Intussusception | 2.416 | 1.122 | 11.200 | 0.031 |
| Enhancement pattern | -0.883 | 0.461 | 0.413 | 0.055 |
| Enhancement level | -1.524 | 0.363 | 0.218 | <0.001 |
| Enhancement level | -0.883 | 0.461 | 0.413 | 0.055 |
| Adjacent peritoneum | 1.435 | 0.479 | 4.200 | 0.003 |
| Loco-regional lymph node | 2.620 | 0.547 | 13.74 | <0.001 |

**Supplementary Table 2** Arterial CT texture parameters between non-lymphoma group and lymphoma group in patients with primary small bowel malignancies

| Parameters | Non-Lymphoma  (n = 57) | Lymphoma  (n = 30) | p value |
| --- | --- | --- | --- |
| Correlation_AllDirection_offset1_SD (×10^-9) | 13.35±2.86 | 48.15±15.44 | <0.001 |
| ClusterProminence_angle135_offset1 (×10^6) | 41.89±4.10 | 18.81±1.95 | <0.001 |
| ClusterProminence_AllDirection_offset7_SD (×10^11) | 182.82±46.21 | 26.31±9.35 | <0.001 |
| ClusterShade_AllDirection_offset4_SD (×10^7) | 19.16±4.54 | 5.93±1.94 | <0.001 |
| RunLengthNonuniformity_AllDirection_offset4_SD | 412.54±92.32 | 1484.38±617.66 | <0.001 |
| ClusterShade_angle90_offset4 (×10^3) | 37.11±9.13 | -12.34±5.06 | <0.001 |
| HaralickCorrelation_AllDirection_offset4_SD (×10^12) | 113.23±28.97 | 75.81±64.02 | <0.001 |
| Maximum3Ddiameter | 58.84±3.02 | 80.14±6.26 | 0.001 |

Note: The data are presented as average ± standard deviation.

**Supplementary Table 3** Venous CT texture parameters between non-lymphoma group and lymphoma group in patients with primary small bowel malignancies

| Parameters | Non-Lymphoma (n = 57) | Lymphoma  (n = 30) | p value |
| --- | --- | --- | --- |
| GreyLevelNonuniformity_AllDirection_offset4_SD (×10^-2) | 3.55±1.78 | 5.11±0.89 | <0.001 |
| RunLengthNonuniformity_AllDirection_offset4_SD | 340.12±67.33 | 1162.38±499.30 | 0.001 |
| MajorAxisLength | 59.49±3.35 | 81.21±6.45 | 0.001 |
| HaralickCorrelation_AllDirection_offset4_SD (×10^12) | 98.63±19.25 | 43.99±25.77 | 0.001 |
| Percentile90 | 117.78±4.81 | 97.03±1.84 | 0.002 |
| Correlation_AllDirection_offset1_SD (×10^-10) | 125.03±21.67 | 310.92±83.60 | 0.002 |
| HighGreyLevelRunEmphasis_AllDirection_offset4_SD | 315.32±66.77 | 94.65±14.60 | 0.007 |
| Correlation_angle45_offset4 (×10^-4) | 2.67±0.22 | 1.97±0.38 | 0.010 |

Note: The data are presented as average ± standard deviation.

**Supplementary Table 4**. The diagnostic performance of arterial texture features in distinguishing the non-lymphoma and lymphoma of the small bowel

| Parameter | Cut-off | Acc | Sen | Spe | PPV | NPV |
| --- | --- | --- | --- | --- | --- | --- |
| Correlation_AllDirection_offset1_SD | -0.230 | 0.744 | 0.800 | 0.714 | 0.600 | 0.870 |
| ClusterProminence_angle135_offset1 | -0.288 | 0.744 | 0.767 | 0.732 | 0.605 | 0.854 |
| ClusterProminence_AllDirection_offset7_SD | -0.383 | 0.733 | 0.800 | 0.696 | 0.585 | 0.867 |
| ClusterShade_AllDirection_offset4_SD | -0.364 | 0.698 | 0.767 | 0.661 | 0.548 | 0.841 |
| RunLengthNonuniformity_AllDirection_offset4_SD | -0.297 | 0.628 | 0.900 | 0.482 | 0.482 | 0.900 |
| ClusterShade_angle90_offset4 | -0.003 | 0.674 | 1 | 0.500 | 0.517 | 1 |
| HaralickCorrelation_AllDirection_offset4_SD | -0.221 | 0.640 | 0.933 | 0.482 | 0.491 | 0.931 |
| Maximum3Ddiameter | -0.362 | 0.651 | 0.867 | 0.536 | 0.500 | 0.882 |

Note: Acc, accuracy; Sen, sensitivity; Spe, specificity; PPV, positive predictive value; NPV, negative predictive value.

**Supplementary Table 5**. The diagnostic performance of venous texture features in distinguishing the non-lymphoma and lymphoma of the small bowel

| Parameter | Cut-off | Acc | Sen | Spe | PPV | NPV |
| --- | --- | --- | --- | --- | --- | --- |
| GreyLevelNonuniformity_AllDirection_offset4_SD | -0.248 | 0.733 | 0.767 | 0.714 | 0.590 | 0.851 |
| RunLengthNonuniformity_AllDirection_offset4_SD | -0.204 | 0.733 | 0.667 | 0.768 | 0.606 | 0.811 |
| MajorAxisLength | -0.549 | 0.605 | 0.933 | 0.429 | 0.467 | 0.923 |
| HaralickCorrelation_AllDirection_offset4_SD | -0.450 | 0.698 | 0.700 | 0.696 | 0.553 | 0.812 |
| Percentile90 | -0.033 | 0.686 | 0.967 | 0.536 | 0.527 | 0.968 |
| Correlation_AllDirection_offset1_SD | -0.116 | 0.709 | 0.600 | 0.768 | 0.581 | 0.782 |
| HighGreyLevelRunEmphasis_AllDirection_offset4_SD | 0.013 | 0.558 | 0.967 | 0.339 | 0.439 | 0.950 |
| Correlation_angle45_offset4 | -0.560 | 0.686 | 0.533 | 0.768 | 0.552 | 0.754 |

Note: Acc, accuracy; Sen, sensitivity; Spe, specificity; PPV, positive predictive value; NPV, negative predictive value.

**Supplementary Table 6**. The multivariate logistic regression analysis of arterial texture features

|  | Odds Ratio (95% CI) | p value |
| --- | --- | --- |
| Correlation_AllDirection_offset1_SD | 53.70 (2.41-1194.66) | 0.012 |
| RunLengthNonuniformity_AllDirection_offset4_SD | 4.86 (0.44-53.90) | 0.197 |
| ClusterShade_AllDirection_offset4_SD | 0.11 (0.01-1.10) | 0.061 |
| ClusterShade_angle90_offset4 | 0.01 (0.00-0.14) | 0.001 |
| HaralickCorrelation_AllDirection_offset4_SD | 3.00 (0.96-9.38) | 0.058 |
| ClusterProminence_AllDirection_offset7_SD | 0.06 (0.00-23.48) | 0.360 |

**Supplementary Table 7**. The multivariate logistic regression analysis of venous texture features

|  | Odds Ratio (95% CI) | p value |
| --- | --- | --- |
| GreyLevelNonuniformity_AllDirection_offset4_SD | 0.40 (0.09-1.77) | 0.227 |
| RunLengthNonuniformity_AllDirection_offset4_SD | 24.97 (1.72-362.92) | 0.018 |
| Percentile90 | 0.33 (0.12-0.91) | 0.032 |
| Correlation_AllDirection_offset1_SD | 2.78 (1.18-6.53) | 0.019 |
| HighGreyLevelRunEmphasis_AllDirection_offset4_SD | 0.23 (0.02-2.37) | 0.216 |
| Correlation_angle45_offset4 | 0.36 (0.17-0.77) | 0.009 |

**Supplementary Table 8**. The diagnostic performance of SVM classifier and logistic regression model

|  | Arterial Texture | |  | Venous Texture | |
| --- | --- | --- | --- | --- | --- |
|  | SVM | Logistic regression |  | SVM | Logistic regression |
| Accuracy | 0.895 | 0.872 |  | 0.930 | 0.814 |
| Sensitivity | 0.967 | 0.833 |  | 1 | 0.733 |
| Specificity | 0.857 | 0.893 |  | 0.893 | 0.857 |
| PPV | 0.784 | 0.804 |  | 0.833 | 0.730 |
| NPV | 0.980 | 0.910 |  | 1 | 0.859 |

Note: PPV, positive predictive value; NPV, negative predictive value.
